# *Let us be heard*: critical analysis and debate of collaborative research approaches used in implementation science research with equity-deserving populations

**DOI:** 10.1186/s43058-025-00695-z

**Published:** 2025-01-24

**Authors:** Sarah Madeline Gallant, Cynthia Mann, Britney Benoit, Megan Aston, Janet Curran, Christine Cassidy

**Affiliations:** 1 Dalhousie University, Halifax, NS Canada; 2 IWK Health and Dalhousie University, Halifax, NS Canada; 3 Saint Francis Xavier University, Antigonish, NS Canada

**Keywords:** Collaborative research approaches, Integrated knowledge translation, Marginalized populations, Substance use disorder, Implementation research

## Abstract

**Background:**

Implementation Science research completed with equity-deserving populations is not well understood or explored. The current opioid epidemic challenges healthcare systems to improve existing practices through implementation of evidence-based interventions. Pregnant persons diagnosed with opioid use disorder (OUD) is an equity-deserving population that continues to experience stigmatization within our healthcare system. Efforts are being made to implement novel approaches to care for this population; however, the implementation research continues to leave the voices of pregnant persons unheard, compounding the existing stigma and marginalization experienced.

**Methods:**

This debate paper highlights a specific case that explores the implementation of the Eat, Sleep, Console (ESC) model of care, a function-based empowerment model used to guide the care for pregnant persons diagnosed with OUD and their infants. We establish our debate within the conceptual discussion of Nguyen and colleagues (2020), and critically analyze the collaborative research approaches, engaged scholarship, Mode 2 research, co-production, participatory research and IKT, within the context of engaging equity-deserving populations in research. We completed a literature search in CINAHL, Google Scholar, PubMed and Embase using keywords including collaborative research, engagement, equity-deserving, marginalized populations, birthparents, substance use and opioid use disorder with Boolean operators, to support our debate.

**Discussion:**

IKT and Community Based Participatory Action Research (CBPR) were deemed the most aligned approaches within the case, and boast many similarities; however, they are fundamentally distinct. Although CBPR’s intentional methods to address social injustices are essential to consider in research with pregnant persons diagnosed with OUD, IKT aligned best within the implementation science inquiry due to its neutral philosophical underpinning and congruent aims in exploring complex implementation science inquiries. A fundamental gap was noted in IKT’s intentional considerations to empowerment and equitable engagement of equity-deserving populations in research; therefore, we proposed informing an IKT approach with Edelman’s Trauma and Resilience Informed Research Principles and Practice (TRIRPP) Framework.

Contributions to the literature

- Implementation research completed with equity-deserving populations is not well understood or explored.
- Integrated Knowledge Translation is a collaborative approach founded within implementation science; however, it does not highlight explicit considerations of empowerment and engagement with equity-deserving populations.
- This paper describes tangible methods to mitigate the gap in intentional consideration of equitable engagement and empowerment present in using an IKT approach to implementation science research by informing an IKT approach with the TRIRPP framework.
- IKT informed by TRIRPP can support researchers in collaboratively exploring implementation inquiries with equity-deserving populations.

## Background

Implementation science is defined as “the scientific study of methods to promote the systematic uptake of research findings and other EBPs [evidence-based practices] into routine practice, and, hence, to improve the quality and effectiveness of health services (p.1)” [1]. Implementation science at its core boasts collaborative and practical nuances. The general aims of implementation science inquiries are to effectively move findings into practice, which requires communication, empowerment and deliberations with the people who will be using this knowledge, such as knowledge users [2]. Research completed with equity-deserving populations presents unique considerations for implementation science researchers. Equity-deserving populations can be defined as “a group of people who, because of systemic discrimination, face barriers that prevent them from having the same access to the resources and opportunities that are available to other members of society, and that are necessary for them to attain just outcomes (p.1) [3]”. Researchers must complete ethical and caring research as our duty to care with individuals and communities of equity-deserving populations [4]. Researchers should always conduct research in a collaborative way, especially with those who could be trauma-exposed, such as pregnant persons diagnosed with opioid use disorder (OUD). Jefferson and colleagues (2021) completed a scoping review of recommendations available to support research with trauma-exposed individuals. Key findings suggest populations who are marginalized and stigmatized should not be excluded from research. Exclusion perpetuates the feelings of disempowerment found in the healthcare system; therefore, researchers must empower pregnant persons diagnosed with OUD as equal partners in healthcare research [5].

Collaborative research approaches, in the most basic form, are approaches used in research that involve collaboration between researchers and knowledge users, including institutions, organizations and communities [6]. Collaborative research is founded on developing partnerships and clear communication between researchers and knowledge users, with a shared aim to create high-quality and relevant research findings [6, 7]. Integrated knowledge translation (IKT) is a commonly used collaborative research approach in implementation science [7]. Kothari and colleagues (2017) define IKT as “a model of collaborative research, where researchers work with knowledge users who identify a problem and have the authority to implement the research recommendations (p.299)” [8]. To develop conceptual clarity, Nguyen and colleagues (2020) completed an inquiry comparing IKT to four popular collaborative research approaches (engaged scholarship, Mode 2 research, co-production, and participatory research). Although this inquiry provides important insight, it did not directly focus on approaches used with equity-deserving populations. As such, questions remain if IKT is the most appropriate collaborative research approach in completing implementation research with equity-deserving populations. This debate article will explore a research case examining an implementation science inquiry with an equity-deserving population. We will examine popular collaborative research approaches and provide a contextual discussion on which research approach is best suited for implementation science inquiries with equity-deserving populations.

### The research case

Birthparents diagnosed with substance use disorder (SUD), including opioid use disorder and their infants diagnosed with neonatal abstinence syndrome (NAS)/ neonatal opioid withdrawal syndrome (NOWS) are equity-deserving populations that have historically been stigmatized and dehumanized in our healthcare system [9–12]. “Lethal fetal containers (p.17)” [9] and “Heroin Birthparents or Methadone Babies (p.127)” [13] are only a handful of examples of the stigmatized and discriminatory language that has been used in society and the healthcare system. Broadly, the incidence of NAS across the globe has demonstrated a substantial increase in the last decade [14]. For example in British Columbia, Canada incidence rates of newborns diagnosed with NAS have nearly doubled in the last decade (2010–2020) from 2.6 to 4.8 per 1,000 live births, respectively [15]. This substantial growth challenges the already taxed healthcare system, as complexities are found within the care for birthparents and their babies, often stemming from cyclical and generational impacts of stigma, trauma, and diverse comorbidities [11, 16, 17].

Implementation of a function-based empowerment model titled the Eat, Sleep, Console (ESC) model of care is a significant evolution in the care for this population. The ESC model empowers birthparents to care for their infants and demonstrates positive patient and health system outcomes, including decreases in length of hospital stay, pharmacological treatment for infants and total hospital costs [18, 19]. The core components of the ESC model include a function-based assessment tool, prioritization and optimization of first line non-pharmacological interventions and most importantly empowerment of the birthparents in the care for their newborns [20]. Despite the promising impacts of the ESC model of care, little is known about how to implement this model into practice effectively. Moreover, studies exploring implementation have left the voices of birthparents unheard, contributing to stigma and marginalization experienced within this population.

### Research with birthparents diagnosed with OUD/SUD

The complex influences of stigma experienced by birthparents diagnosed with OUD in the healthcare system present barriers to completing successful health research with this equity-deserving population. Historically, research has been completed on this population rather than with them. Birthparents seek to be engaged with respect and without judgement, treated as human beings, with their voices heard and valued [11].

Collaborative partnerships in research with birthparents diagnosed with OUD help to (a) address disparities, such as equitable access to research engagement; [21] (b) provide birthparents with authority over their experience; [22] and (c) elevate the voices of birthparents, empowering a sense of control to inform the care they will receive within the healthcare system [21, 23]. Despite the recommendations of collaboration and empowerment, birthparents diagnosed with OUD continue to remain unseen in healthcare research [9, 23], specifically in research exploring the implementation of the ESC model of care. As such, this paper will critically analyze and debate collaborative research approaches in implementation science using this research case as an example.

## Methods

Our debate is established within the conceptual discussion of Nguyen and colleagues (2020), which explored collaborative research approaches in relation to integrated knowledge translation (IKT), the most widely used collaborative research approach in implementation science research. Nguyen and colleagues (2020) qualitative study explored expert opinions and experiences in using various collaborative research approaches, contributing to our conceptual understanding of the different collaborative research approaches available. However, limited discussion is found in Nguyen and colleagues' (2020) inquiry about which collaborative research approach is best suited for research with equity-deserving populations. We searched literature in CINAHL, Google Scholar, PubMed and Embase using keywords including collaborative research, engagement, equity-deserving, marginalized populations, birthparents, substance use and opioid use disorder with Boolean operators, to understand the use of collaborative research approaches with birthparents diagnosed with opioid use disorder. We will debate the following five collaborative approaches outlined in Nguyen and colleagues' (2020) inquiry: Mode 2 Research, Engaged Scholarship, Co-Production, Participatory Research and IKT, in the context of research with equity-deserving populations.

## Discussion

### Mode 2 research and engaged scholarship

We noted the first two methods, Mode 2 research and engaged scholarship, minimally in our search. This finding is consistent with how Nguyen and colleagues (2020) conceptualizes these two approaches. The language describing these two methods is not sensitive or reflective of the inclusive considerations needed to conduct research with equity-deserving populations. Mode 2 research supports collaborative trans-disciplinary partnerships to create knowledge on questions emerging from ‘working life (p.250)’ [24]. The primary aim of Mode 2 research is to develop knowledge within and reflective of context. This method’s primary motivation is to increase the social accountability of research [7, 25]. In our review, Mode 2 research was not used to support engagement in research with equity-deserving populations.

Similar findings were noted with engaged scholarship, another collaborative approach that supports the engagement of key partners to explore complex problems in unison rather than in siloes [7]. Engaged scholarship’s primary motivation is to foster a collaborative relationship between universities and communities, supporting university researchers to reconnect with societal needs [7, 26]. Mode 2 research and engaged scholarship use language to describe their partners as ‘end-users’ or ‘industry’ and ‘stakeholders’ or ‘public members’, respectively [7]. This language is disempowering, widening the gap between researcher and consumer rather than bridging in partnerships. Moreover, both approaches engage partners in research for their expertise to ensure researchers meet objectives identified in their populations; however, neither approach empowers partners or provides reflective space for meaningful engagement with equity-deserving populations [7, 26].

### Co-production approach

Co-production was one of the most used collaborative approaches identified in our review. This finding parallels with the conceptual language described by Nguyen and colleagues (2020). Co-production defined “as a way for academics, practitioners, and patients and the public to work together, sharing power and responsibility across the whole research cycle (p.3)”, [27] aims to develop or deliver with consumers effective and efficient services. Researchers actively engage partners in this collaborative approach depending on their capabilities and interests [7]. Co-production has been used successfully with equity-deserving populations, such as birthparents diagnosed with SUD, supporting empowerment and contributing to birthparents feeling heard [28]. Zisman-Ilani and colleagues (2022) describe a virtual method to guide co-production research with birthparents diagnosed with SUD. Virtual Community Engagement Studio (V-CES) is an interactive method that facilitates co-production through various steps to ensure the empowered engagement of partners in co-production research. In this model of co-production, researchers identify the research theme or topic area. This example highlights how often researchers using a co-production approach identify the inquiry rather than it be an inquiry identified from within the equity-deserving population [28]. Supporting equity-deserving populations to identify inquiries contributes to positive participant engagement, empowerment and feelings of being heard [29]. In addition, although co-production is a potential method for collaborating in the presented case, this method has the potential to perpetuate the inequities experienced by birthparents due to the potential structural barriers. The time-bound nature of the case’s inquiry (a PhD inquiry) could potentially create additional inequities by not providing co-producers with sufficient time to engage meaningfully [30].

### Integrated knowledge translation and participatory action research

**Fig. 1:**
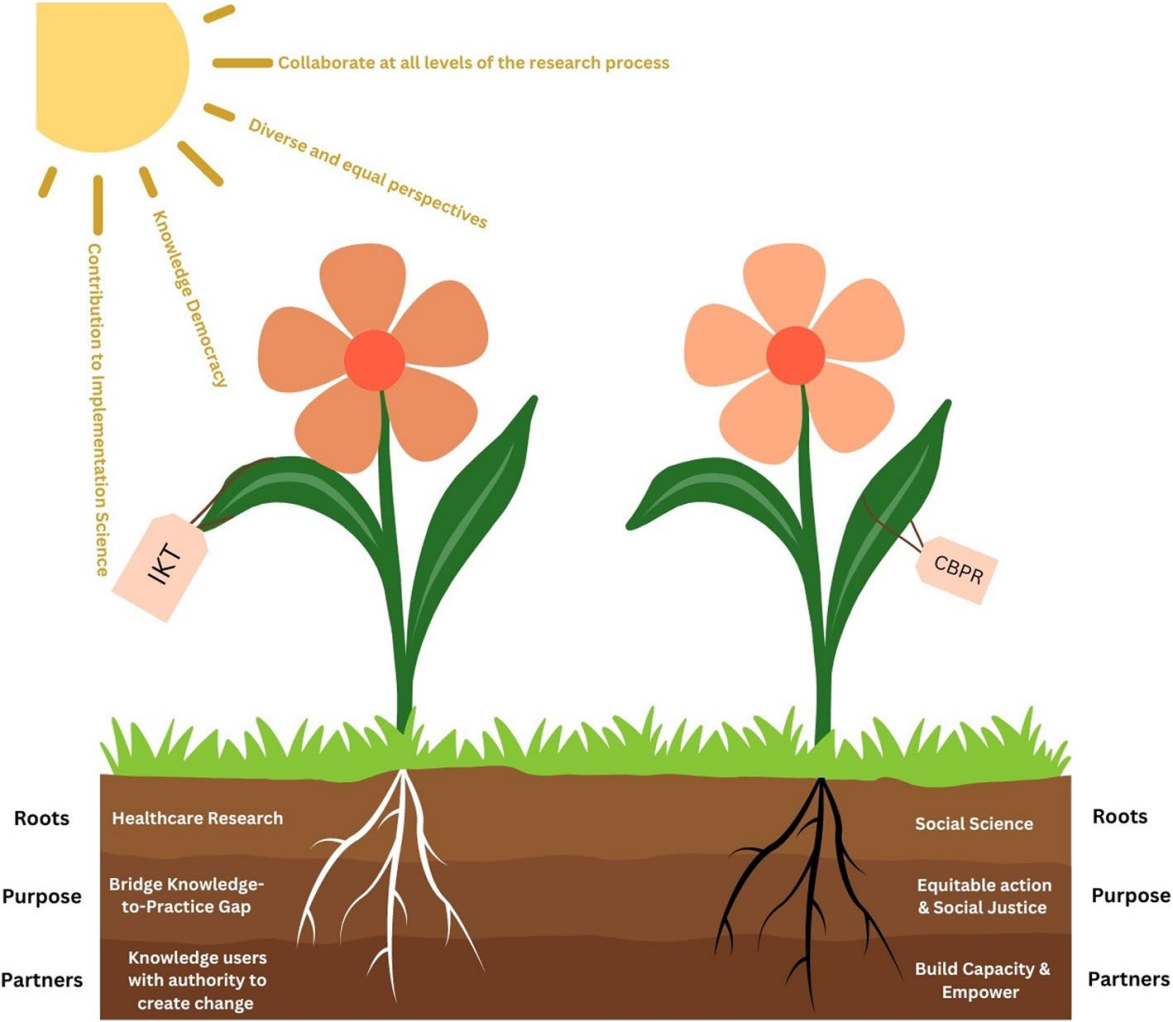
Demonstrates surface commonalities between IKT and CBPR, along with foundational differences present between collaborative approaches

As mentioned previously, IKT has been used widely in implementation science; however, our literature search revealed that IKT’s use as a collaborative research approach for equity-deserving populations has been limited. IKT has been used to conduct research with equity-deserving populations such as sex workers [31], youth experiencing violence [32] and children diagnosed with mental health disorders [33]; however, in our scan, we noted participatory action research (PAR) to be the most prominent collaborative approach to research with equity-deserving populations. The conceptual discussion of PAR in relation to IKT by Nguyen and colleagues highlights similar findings [7]. PAR aims to explore inquiries arising from equity-deserving communities in a collaborative and empowering way [7, 34]. Research studies with birthparents diagnosed with SUD have successfully used PAR as the philosophical underpinnings and methodological aims of PAR, including developing trusting and meaningful relationships, parallel with the identified research needs of birthparents diagnosed with SUD [35]. Figure 1 demonstrates a visual representation of surface commonalities and foundational differences between IKT and PAR, specifically community-based participatory action research (CBPR), as discussed below.

### Integrated knowledge translation

IKT is a collaborative research approach rooted in healthcare research [7]. First conceptualized by Graham in 2007, IKT has gained popularity in healthcare research [7, 36]. IKT grounds itself in a philosophically neutral stance, allowing its dynamic applicability with a wide variety of methodologies used in health research [7]. The primary aim of IKT is to address the knowledge-to-practice gap by developing applicable knowledge that can directly impact change in the healthcare system [37, 38]. Partnerships are central to IKT, where expertise from researchers and key knowledge users, such as healthcare providers, patients, or health system leaders, are equally valued and seamlessly integrated to create relevant knowledge [34, 39, 40].

### Participatory action research

PAR is a collaborative research approach and methodology that originates in social sciences, focusing on community-identified issues [7]. This research approach boasts the largest body of literature and dates back to the 1940s. Developed to address the injustices present in research, the Southern tradition (Freire 1970) aligns with PAR’s most widely used version, CBPR [7]. A diverse set of underpinnings provides a foundation for CBPR; [7] however, the fundamental epistemological belief of CBPR is that knowledge is developed in collaborative social relationships [41]. Empowerment and capacity building are essential to using a CBPR approach to research, as this approach seeks power and emancipation for equity-deserving populations in research and healthcare [7, 34, 42]. This approach begins within the community and provides principles and formalized structures to ensure meaningful participation in research [43]. The power dynamics innately found between researcher and participant are given special consideration in CBPR. CBPR de-centers research expertise and emphasizes the importance and legitimacy of community knowledge. This approach empowers community members to have the authority to control research, influencing and creating change [34, 42].

### Commonalities between IKT and CBPR

On the surface, IKT and CBPR are terms that have been used interchangeably due to the numerous similarities noted between these two collaborative approaches. Similarities include (1) the importance of collaboration at all levels of the research process [34, 39, 44], (2) the inclusion of diverse perspectives, valuing the expertise of partners as equals in addressing a research inquiry [7, 34, 39], and (3) the knowledge co-created and guided by these approaches represents “knowledge democracy (p.7)” [34]; where researchers integrate community knowledge and researcher expertise equally to inform and enact change [7, 34, 39]. The co-creation of knowledge, which is reflective of research findings and interpretive influences from researchers, communities and the context in which they originate, is the pinnacle of commonalities for these two approaches [34, 39, 40, 42].

### Points of difference

The interchangeability of IKT and CPBR is often misinformed. Although many similarities exist at the surface level, they have distinct differences. Most notable are the differences in their foundational roots, their purpose and their partnerships formed. Reflecting on these distinctions is imperative to ensure the appropriate selection of the approach that best meets the needs of the proposed case inquiry [34].

#### Foundational roots

IKT is the only collaborative research approach that originates from within the healthcare context in response to the knowledge-to-practice gap, where knowledge development and translation in practice are inadequate to meet the needs of the growing population [7, 37]. IKT’s foundational roots within the healthcare system facilitate its ability to explore complex contextual health inquiries. Although IKT’s roots are not in emancipation or social justice, it does offer the ability to explore topics within equity-deserving populations. IKT takes a philosophically neutral stance, which lends to its ability to be philosophically pragmatic, adjusting to the philosophy underpinning the study it is being applied to [7]. The pragmatic neutrality of IKT parallels the ways of knowing within the health discipline. For instance, Benoit and Unsworth [31] completed an inquiry with sex workers in Canada. In their IKT-led study, they adjusted their initial aim of mobilizing knowledge on criminal justice to a more dynamic focus on occupational and social rights for sex workers in Canada [31]. The philosophical neutrality of IKT allows researchers to adjust their inquiry based on the dynamic needs of the population.

CBPR, on the other hand, originates within social sciences, with roots in justice, power and emancipation [7]. CBPR is embedded in ‘action research’ founded by Kurt Lewin (social scientist) [45]. Participation is the foundational building block for all action research, including CPBR, where its ontological and epistemological beliefs embrace a participative reality and way of knowing [45]. For example, CBPR is more aligned with research questions with roots in emancipation, social injustice and power imbalances, such as Raynor and colleagues' (2022) inquiry exploring the feasibility of a digital intervention to support parenting for birthparents diagnosed with SUD [35]. CBPR was most appropriate for this inquiry as themes, such as historical power imbalances, health inequities to accessible programs and stigmatization, underpinned the research inquiry [35].

#### Purpose

An IKT approach aims to bridge the knowledge-to-practice gap, developing applicable knowledge that is relevant and directly applicable to create an impact in the population affected by the inquiry [37]. An IKT approach aims to use a dynamic and iterative process that incorporates influences from key knowledge users affected by research, valuing expertise from researchers and key knowledge users equally [34, 39, 40]. Although an IKT approach has the potential to mitigate power imbalances, contribute to the empowerment of knowledge users, and emancipate knowledge, these outcomes are secondary to the immediate aim of IKT [7, 8, 34]. For example, Benoit and Unsworth [31] emphasize how IKT supports an end goal to bridge the “know-do” gap that is reflective of research questions and solutions co-created and identified in all aspects of the research process [31].

In contrast, CBPR has three core aims: (1) social and environmental justice with a desire for impactful change, (2) moving knowledge into equitable action; and (3) building capacity or self-determination [7, 46]. CPBR’s intentions move beyond co-creating knowledge with participants and strive to work with communities through research to eliminate social inequities and build their capacity to improve their abilities [43, 44]. For example, Raynor’s (2022) CBPR study aimed to empower birthparents to engage in a research project to design a digital recovery support reflective of the unique needs of their community. A CBPR approach guided these authors to explore community-identified recommendations to support the development of the digital intervention. Although integrated with previous literature, community-identified solutions guided the intervention development [35].

It is important to note that although both approaches aim to co-create knowledge, CBPR values community-identified solutions rather than research-based solutions [40]. This is often a point of discourse between IKT and CBPR as IKT values the equal co-creation of knowledge that incorporates expertise from researchers and communities and identifies interventions and practice changes based on research findings [39].

#### Partners

The most distinct difference between the two approaches is how they partner with their communities. IKT uses the term “knowledge users” to describe members of the community who engage with the research team in equal, mutually beneficial partnerships [7, 40]. IKT strives to create a shared perspective between researcher and knowledge user, where both perspectives are valued and heard [39]. IKT’s approach to partnership development is intentional and purposeful. Researchers strategically choose individuals to be engaged in the IKT process who have the authority to create change within the healthcare system [39]. Having decision-makers as essential team members is a unique requirement for IKT. It ensures the knowledge-to-practice gap is addressed as knowledge users selected have the authority to implement findings [37]. For example, Benoit and Unsworth [31] strategically developed their research team encompassing researchers, sex workers and sex worker organizations, as these individuals had the authority to create change. Participants share that by engaging in an IKT approach, the research team was able to focus on a priority-identified question, closing the know-do gap by developing relevant and responsive findings easily implemented by participants to create change [31].

CBPR, like IKT, creates partnerships to improve the relevancy and impact of research findings that reflect the community they impact [43]. However, what is unique to CBPR is that it moves beyond creating partnerships and seeks to build capacity and empower communities through their involvement in the research process. Although IKT might create these opportunities indirectly, CBPR explicitly aims to empower and build capacity within its partnerships [37, 42–44]. CBPR empowers community members to have authority over the research process, directly influencing change within their community [34, 42]. Furthermore, CBPR goes beyond simply addressing power imbalances historically present between researchers and the community as it attempts to de-center research expertise, emphasizing community expertise [34, 43]. These aims parallel CBPR’s commitment to eliminating injustices and inequities in equity-deserving populations. For example, in Raynor and colleagues' (2022) CBPR study, authors developed a comprehensive community advisory board (CAB), including birthparents who achieved long-term recovery. Raynor and colleagues empowered and built capacity within birthparents to engage in research, tailoring interventions based on their expertise (2022).

### Application to the research case

The proposed case for this debate aims to examine the implementation of the ESC model of care. Central to its implementation is the empowerment of birthparents to be the primary decision-makers for their infants as part of a dynamic and collaborative healthcare team [18, 47, 48]. It is imperative to have birthparents’ voices heard in exploring implementation, given the depth of their role in caring for their infants within this model. Historically, birthparents’ voices have been silenced in research and in the care for their infants [16, 23]. Multileveled stigma and discrimination continue to plague the experiences of birthparents [11].

IKT and CBPR are two collaborative research approaches that have the potential to support the inclusive and empowered engagement of birthparents diagnosed with OUD in this inquiry. Although CBPR’s intentional methods to support empowerment, capacity building, and address social injustices are essential to consider in research with birthparents diagnosed with OUD, we would argue IKT is the most appropriate approach for this specific inquiry for the following reasons. First, the case inquiry aims to develop applicable knowledge to support the effective implementation of the ESC model of care, an evidence-based intervention (EBI). This inquiry, stemming from implementation science, aligns with IKT’s primary aim to address the knowledge-to-practice gap by co-creating applicable knowledge to effectively address questions or, in this case, support implementation of an EBI in the healthcare system [37, 39]. Although CBPR also shares the aim of addressing the knowledge-to-practice gap, it comes secondary to the aim of advancing social justice and creating social change [34].

Second, a neutral philosophy underpins IKT, which allows for the seamless integration of IKT with the need for a mixed methods approach to address the case inquiry. Given the complex nature of healthcare delivery, a uniform research approach is often insufficient to holistically answer dynamic research questions in implementation research [49]. A mixed methods approach is imperative in examining the outcomes of an intervention and the ‘how’ or the ‘why’ an intervention was a success or failure in clinical practice [50]. The use of mixed methods as a research approach is often challenged by the competing philosophical underpinnings of quantitative and qualitative research [51, 52]; therefore, having a collaborative research approach that is philosophically neutral and pragmatic, such as IKT, ensures seamless integration of multiple methods within the study.

Finally, this research inquiry originates from within the healthcare system. IKT has been designed to address the contextual aspects unique to complex healthcare inquiries [37]. IKT’s intentional approach to developing partnerships with key knowledge users who have the authority to create impact parallels the aim of the case to develop applicable knowledge to directly inform the implementation and sustainment of the ESC model of care [34]. Furthermore, healthcare is evidence-based and is subject to scrutiny; therefore, it requires empirically supported interventions that are culturally and community based rather than solely community or empirically based [53]. IKT shares this value, emphasizing the importance of identifying interventions supported by research [39].

Despite the numerous aspects of IKT that align with this case, IKT presents with one significant limitation. IKT does not encompass purposeful methods to ensure meaningful and empowered engagement of equity-deserving populations in its approach. Although empowerment and capacity building are often secondary outcomes in using an IKT approach, we argue that these outcomes should intentionally be part of IKT’s partnership formation when conducting research with equity-deserving populations. Kothari and Wathen [39] also agree, stating the importance of IKT to apply purposeful methods to ensure meaningful engagement of key knowledge users in all aspects of the research process. For this reason, we propose using Edelman’s [54] Trauma and Resilience Informed Research Principles and Practice (TRIRPP) Framework to intentionally empower, build capacity and meaningfully engage birthparents diagnosed with OUD.

### TRIRPP

Birthparents diagnosed with OUD have complex and often trauma-exposed histories that are perpetuated by the cyclical and ongoing stigmatization within the healthcare system and research [11, 16, 21]. Given this history, a trauma-informed approach to research is needed to reduce disparities and avoid re-traumatization [16, 17, 21]. Edelman [54] presents the Trauma and Resilience Informed Research Principles and Practice (TRIRPP) Framework as a guide to support the inclusive, empowered, and safe participation of equity-deserving and trauma-exposed populations in health research. TRIRPP encompasses eight principles, reflecting the thoughtful integration of trauma and resilience-informed practice principles. Informing an IKT approach with the TRIRPP Framework can support inclusive, safe and empowered research with birthparents diagnosed with OUD in the case inquiry [54]. Underpinning an IKT approach with the TRIRPP framework encourages researchers to intentionally consider, reflect and act to advance equity and inclusion in completing research with equity deserving populations Table 1 highlights each of the eight principles of the TRIRPP framework and how they can be integrated within an IKT approach to research. For example, principle one “Actively seek out participation from equity-deserving populations” can be operationalized by being continuously reflective of the use of inclusive and empowering language in written and verbal communication.

**Table 1:** Demonstrates the TRIRPP framework’s eight principles and integration with an IKT approach to the case inquiry [54]

	**Principle**	**Example of principles within an IKT approach**
1	Actively seek out participation from equity-deserving populations	• Partner with representatives within equity-deserving populations as key knowledge users • Use language that is conducive to power sharing and empowerment
2	Take a social justice stance: address queries of deprivation and health inequities	• Ensure adequate reflection of the impact of social inequality • Incorporate reflexivity amongst all members of the research team (key knowledge users) • Ensure research questions are reflective of practices as change agents rather than individuals
3	Ensure partnerships are framed as “relationships”	• Creating a trusting and committed relationship to ensure inclusive and safe research is completed • Provide space for individuals within the research team to meet and build their interconnected relationships • Ensure partners are aware they can withdraw at any point
4	Empower partnerships through choice and agency	• Discussing the process of data collection with the community advisory board (CAB) to determine a protocol that best supports empowerment and agency (i.e. participants begin the recording when they feel ready) • Ongoing and established periodic discussions of informed consent and involvement with data collection and research conduction • A co-creation approach must be foundational to knowledge creation, ensuring key knowledge users are empowered to share their perspectives that are equally valued
5	Emphasize strengths and resilience within partnerships	• Engage with active listening in all encounters with birthparents diagnosed with OUD • In the initiation phase of the IKT approach, provide space for birthparents to share their experiences of trauma within the healthcare system and how that impacts the relationships they are forming on the CAB
6	Avoid re-traumatization (address)	• Create an open dialogue on trauma with CAB members to discuss concepts such as language to be used and questions to be asked • Include birthparents in all aspects of the research process, including technical aspects such as protocols for data protection
7	Recognize the impact of trauma on all participants	• Develop established supports (emotional and social support) that can be offered to all participants regardless of their population of origin • Create data collection protocol (including location for interviews, set up of interview space, individuals included in the interview, etc.) with birthparents diagnosed with OUD
8	Seek to provide safety and cultural competence in research	• Create a diverse CAB that is representative of the populations included • Create dynamic research questions that have the potential to uncover cultural impacts on implementation

### IKT and TRIRPP

IKT is a dynamic and flexible approach to complete collaborative research. Absent of prescription, this approach allows researchers to engage in the core stages and principles of IKT as needed to complete their proposed study with integrity [37]. IKT has two general phases: (1) the initiation phase and (2) the application of the theory phase. Principles from the TRIRPP framework can be used to intentionally acknowledge the complex histories and social inequities found within equity-deserving populations, while engaging in an IKT approach. Moreover, this framework recognizes power imbalances and the importance of empowering individuals as integral knowledge users within the case inquiry [54]. The eight principles of the TRIRPP framework will form a foundation for conducting research with birthparents diagnosed with OUD, emphasizing critical points for reflexivity and important considerations for engagement with participants. The TRIRPP principles will encourage intentional considerations of the impacts of trauma and ways to mitigate re-traumatization for birthparents diagnosed with OUD [54]. Although all principles are present throughout the entire research process in various ways, we have outlined below examples of where we can explicitly integrate them into the case inquiry.

#### Initiation phase

In the initiation phase of an IKT approach, researchers develop foundational relationships, communication plans, determine clear roles, and create space to address power imbalances and the accessibility of the research engagement [37, 55, 56]. In the research case, principles one through four of the TRIRPP framework are imperative to apply to ensure intentional reflection and acknowledgement of the historical trauma and stigma that birthparents diagnosed with OUD have faced [54]. S.Gallant (Author 1) has been immersed in the field as a researcher and care provider with birthparents diagnosed with SUD since 2017. The long-term immersion and dialogue that S.Gallant has been privileged to has allowed her to develop trusting relationships reflecting respect and commitment. The reciprocal relationships developed ensure adequate attention is given to the inequities present in this population [37]. For example, S.Gallant has learned that birthparents diagnosed with SUD often face barriers in engaging in research due to structural barriers such as access to transportation or childcare; therefore, we recommend in this case inquiry to create space in initial conversations to ensure structural barriers and inequities present in access to engagement in research are acknowledged and addressed collaboratively by the research team.

We recommend engagement with birthparents diagnosed with OUD as part of a community advisory board (CAB) within this case (principle 1). This advisory board should encompass all key knowledge users with the authority to create impact and change within the healthcare system, including healthcare providers (physicians, nurses), health system leaders (including managers), and birthparents diagnosed with OUD. A diverse CAB and the potential for power imbalances requires a relational approach to partnership development to create a safe and inclusive environment for the engagement of birthparents diagnosed with OUD (principle 3). We recommend explicitly acknowledging that everyone is an equal partner on the research team, emphasizing the valuable and unique contributions that all decision-makers bring to the research inquiry. This open dialogue will provide space for discussions on power dynamics, clear roles, and responsibilities, ultimately empowering individuals through their engagement in co-creating research processes and outcomes (principles 4 and 5).

The remaining principles of the TRIRPP framework will underpin movement through the research process. The CAB will iteratively influence decisions throughout the research study, intentionally empowering birthparents diagnosed with OUD to inform integral aspects of the research process. In the data collection stage especially, TRIRPP will inform safe and caring environments for interviewing birthparents diagnosed with OUD. We recommend that interview guides and techniques should be informed by the CAB, with special considerations to acknowledge the impacts of trauma and avoid re-traumatization (principles 2, 6, and 7). Data analysis should incorporate the same principles to ensure (1) adequate representation of findings, (2) a holistic understanding of the birthparent’s experience with the implementation of the ESC model of care, and (3) careful attention to the key contextual aspects influencing implementation success or failure. This approach will ensure that findings are culturally relevant and reflective of critical health inequities needing to be addressed [54].

#### Application of theory phase

The second phase of IKT is to move the knowledge co-created by researchers and key knowledge users into practice in an applicable way. A framework, such as the knowledge-to-action (KTA) framework, typically guides this phase of an IKT approach. The KTA framework has two cycles: the knowledge creation stage and the action cycle [57]. The knowledge creation phase of the cycle is represented above in the initiation phase of IKT [58]. We recommend co-creating knowledge on implementation outcomes of the ESC model of care that reflects diverse and culturally sensitive perspectives. Incorporating periods of reflexivity to determine how implementation outcomes reflect social inequities, will add depth to the case inquiry. Engaging in periods of reflexivity will also create intentionality in this case inquiry to ensure social injustices are considered in the implementation of the ESC model of care for birthparents diagnosed with OUD and their infants (principle 2).

The second phase of the KTA cycle is the action cycle, which is the deliberate and intentional application of knowledge to inform clinical practice [58]. The TRIRPP framework can also influence this phase of the KTA cycle. The action cycle is an iterative process that addresses local facilitators and barriers to knowledge translation of findings, including implementing, evaluating, and sustaining knowledge in practice [58]. The TRIRPP framework will support an intentional consideration for equitable translation of findings. The knowledge co-created in phase one of this IKT project will then be directly implemented in the healthcare system as the individuals included in the CAB have the authority to enact change with the findings; thus, the knowledge to practice gap is bridged, and ultimately, there is potential for healthcare to be improved [39].

## Conclusion

Implementation science research is innately collaborative, requiring engagement from key knowledge users to improve translation of research findings into practice. IKT is a commonly used approach in implementation science research; however, questions exist of if it is the best approach for completing research with equity-deserving populations. Various collaborative research approaches exist including IKT, Mode 2 research, engaged scholarship, co-production and participatory action research (CPBR). Following a tiered literature review and reflective dialogue, integrated knowledge translation (IKT) and community-based participatory action research (CBPR) were deemed the most suited for a research case exploring an implementation science inquiry with an equity-deserving population. Although CBPR’s intentional methods to support empowerment, capacity building, and address social injustices are important to consider in research with equity-deserving populations, an IKT approach shares a parallel aim within implementation science; it provides a neutral philosophical underpinning to allow for a mixed-method investigation, and it originates within a healthcare context, valuing research-based solutions reflective of community perspectives. IKT; however, demonstrates a gap in the intentionality to empower and ensure equitable engagement of equity-deserving populations in research. For that reason, we proposed integrating the Trauma and Resilience Informed Research Principles and Practice (TRIRPP) Framework to address this gap and ensure intentional reflection of equity and empowerment of equity-deserving populations such as birthparents diagnosed with OUD. In using an IKT approach informed by the TRIRPP framework, researchers can empower, include, and create a safe environment for equity-deserving populations to engage in healthcare research.

## Data Availability

Not applicable.

## Abbreviations

OUD: Opioid Use Disorder
ESC: Eat, Sleep, Console
IKT: Integrated Knowledge Translation
CBPR: Community Based Participatory Action Research
TRIRPP: Trauma and Resilience Informed Research Principles and Practice Framework
EBP: Evidence-Based Practices
SUD: Substance Use Disorder
NAS: Neonatal Abstinence Syndrome
PAR: Participatory Action Research
CAB: Community Advisory Board
KTA: Knowledge to Action
